# Uniportal VATS for anatomical lung resection: 15 years of innovation

**DOI:** 10.3389/fsurg.2026.1931138

**Published:** 2026-08-20

**Authors:** Massimiliano Bassi, Diego Gonzalez-Rivas, Jacopo Vannucci, Marco Anile

**Affiliations:** 1 Thoracic Surgery Unit, Department of General Surgery, Surgical Specialties and Anesthesiology, Sapienza University, Rome, Italy; 2 Department of Thoracic Surgery, Shanghai Pulmonary Hospital, School of Medicine, Tongji University, Shanghai, China; 3 Department of Thoracic Surgery and Minimally Invasive Thoracic Surgery Unit (UCTMI), A Coruña University Hospital, La Coruña, Spain; 4 University of Perugia Medical School, Perugia, Italy

**Keywords:** anatomical lung resections, lobectomy, minimally invasive surgeries (MIS), single-port VATS, sleeve resection, uniportal VATS (U-VATS)

## Abstract

The evolution of thoracic surgery has progressively moved toward minimizing surgical trauma while preserving oncological radicality in the treatment of lung cancer. Within this context, uniportal video-assisted thoracoscopic surgery (VATS) has emerged as one of the most significant innovations in minimally invasive thoracic surgery. In fact, uniportal VATS not only represents the evolution of multiportal VATS, with the potential to reduce postoperative pain, chest wall trauma, and recovery time, but has also led to a paradigm shift in thoracic surgery, redefining the limits of minimally invasive pulmonary resection. Initially developed for minor thoracic procedures, the first milestone in its evolution occurred in 2011 with the report of the first uniportal lobectomy. Since then, uniportal VATS technique has rapidly spread and has become an established minimally invasive approach for lung cancer treatment in many centers worldwide. Progressively, this technique has expanded from lobectomy to segmentectomy and from standard anatomical resections to highly complex bronchovascular reconstructions. After 15 years of innovation, uniportal VATS has profoundly transformed the surgical management of lung cancer and continues to redefine the boundaries of minimally invasive thoracic oncology, influencing the evolution of uniportal robotic platforms. This narrative review aims to describe the historical, technical, and conceptual evolution of uniportal VATS for anatomical lung resection over the years, highlighting the milestones that contributed to its global dissemination and current role in thoracic surgical oncology.

## 1 Introduction

The evolution of thoracic surgery over the last few decades has been driven by a constant effort to reduce surgical trauma without compromising oncological radicality. For decades, posterolateral thoracotomy represented the standard access for anatomical pulmonary resection. Although effective, thoracotomy was associated with considerable chest wall trauma, postoperative pain, respiratory impairment, and delayed functional recovery. In the 1990s, video-assisted thoracoscopic surgery (VATS) first challenged thoracotomy by showing that major pulmonary resections could be performed safely in a minimally invasive fashion (1). Starting with a three- or four-port approach, VATS progressively shifted to a bi-portal technique and finally to a single-port (or uniportal) approach, in order to reduce surgical trauma, postoperative pain and paresthesia. The clinical rationale of uniportal surgery is apparently simple: reducing the number of injured intercostal spaces could potentially reduce chest wall morbidity. However, uniportal VATS also represented a further conceptual change: not simply fewer ports, but a different surgical geometry, in which the camera and instruments enter through the same intercostal space and reproduce a direct, sagittal, “open-like” view of the hilum and mediastinal structures.

The modern story of uniportal VATS began with minor thoracic procedures and gradually progressed toward increasingly complex anatomical resections. Rocco and colleagues first reported uniportal VATS wedge pulmonary resections in 2004, demonstrating that single-port thoracoscopic lung surgery was feasible for diagnostic lung biopsy and pneumothorax surgery (2, 3).

Nevertheless, the turning point came in 2011, when Gonzalez-Rivas and colleagues tested the single-port approach to anatomical lobectomy (4). This event drastically transformed thoracic surgery. In just 15 years, uniportal VATS has evolved from being used only for lower lobectomy to all types of lobectomies, from lobectomies to segmentectomies, and from standard resections to increasingly complex procedures, including bronchial sleeve lobectomies, pulmonary artery reconstructions and double-sleeve resections.

Today, uniportal VATS has become one of the standard minimally invasive approaches in many experienced centers worldwide. Its diffusion also influenced robotic thoracic surgery, stimulating the development of uniportal robotic-assisted thoracic surgery (RATS), which attempts to combine the single-incision philosophy of uniportal VATS with the technical advantages of the robotic platforms (5).

This narrative review aims to provide a historical and conceptual overview of the 15-year evolution of uniportal VATS for anatomical lung resection, highlighting the major technical milestones and innovations that have contributed to its worldwide dissemination and current role in thoracic surgical oncology.

## 2 Methods

A literature search was independently performed by two authors (MB and MA) using PubMed/MEDLINE, Embase, Web of Science, and Scopus, including articles published in English from January 1990 to 31 May 2026. The search strategy combined Medical Subject Headings (MeSH), where applicable, and free-text terms as follows: (“uniportal VATS” OR “single-port VATS” OR “single-port RATS” OR “uniportal RATS”) AND (“lobectomy” OR “segmentectomy” OR “lung cancer” OR “bronchial sleeve lobectomy” OR “pulmonary artery reconstruction” OR “double sleeve” OR “non-intubated” OR “tubeless” OR “awake”). Titles and abstracts were screened for relevance to the historical, technical, and clinical evolution of uniportal thoracic surgery, and potentially relevant articles were subsequently assessed in full text. In addition, the reference lists of selected articles were manually screened to identify further relevant publications. Original reports, technical papers, reviews, propensity-matched studies, and systematic reviews were prioritized.

This article is presented as a narrative review. Therefore, its objective was not to systematically pool and analyze the existing literature, but rather to reconstruct the chronological and conceptual evolution of the technique and to summarize the most significant advances in the field.

## 3 Narrative review

### 3.1 The origins: from single-port minor procedures to the first uniportal VATS lobectomy

The modern uniportal VATS era began with the idea that thoracic surgery could be performed safely through a single small intercostal incision. In the earliest applications, the procedure was used for peripheral pulmonary wedge resections, pleural procedures, pneumothorax surgery, mediastinal nodal biopsy, and other relatively limited operations. These early procedures were important because they demonstrated that through a single access incision the surgical feasibility was maintained thanks to an adequate visualization and satisfying instrument maneuverability, achieving a better postoperative recovery. Additionally, the uniportal approach showed reduced postoperative pain and residual paresthesia compared to the three-port VATS technique for pneumothorax, supported the biological rationale of limiting surgical trauma to one intercostal space (3).

At this stage, uniportal VATS was still regarded by many surgeons as a “minor” technique. The idea of performing an anatomical lobectomy through one incision seemed impractical: lobectomy requires sequential dissection of vessels and bronchi and multiportal VATS created triangulation and allowed different angles for stapling and dissection. The uniportal approach gradually challenged this logic.

The turning point came with the first reported uniportal VATS lobectomy by Gonzalez-Rivas and colleagues in 2011 (4). The case was a left lower lobectomy performed through a 4-cm incision in the fifth intercostal space, at the level of the anterior axillary line, without rib spreading. This single-port approach emerged from a progressive transition from conventional three-port VATS to two-port VATS lobectomy described by D'Amico et al. (6, 7): eliminating the second port was therefore a logical, although brave, progression. This milestone operation showed that anatomical hilar dissection, stapling of pulmonary vessels and bronchus, specimen retrieval, and lymph node dissection could be achieved through one thoracoscopic incision without any derogation to oncological purposes.

The subsequent two-year experience from the same group transformed the first case report into an early clinical series. Between 2010 and 2012, 102 major pulmonary resections were performed by the uniportal approach. In the analyzed lobectomy cohort, the mean operative time was approximately 154 min, the median chest tube duration was 2 days, the median length of hospital stay was 3 days, and no 30-day mortality was reported. This series was critical because it suggested that uniportal VATS lobectomy was not merely feasible, but reproducible in experienced hands (8).

### 3.2 The geometrical revolution: why uniportal VATS is not only “one less port”

Uniportal VATS changes the geometry of minimally invasive thoracic surgery. In multiportal VATS, the surgeon works with triangulation: the camera and instruments enter from separate points, generating different visual and working axes. In uniportal VATS, all instruments enter through the same incision and move along a sagittal plane.

Bertolaccini and colleagues described this transformation as a “Copernican revolution” (9). This definition emphasizes this concept: the innovation was not only cosmetic, but it redefined the way to approach lung surgery in minimally invasive surgery. Rather than creating triangulation from outside the chest, the uniportal approach moves the fulcrum of the operation inside the thoracic cavity. This allows the instruments to approach the target in parallel, from a craniocaudal or caudocranial perspective, with a more direct relationship between visual and motor axes (10). This makes the target lesion, the camera, the instruments, and the monitor align in a way that resembles the direct view of open surgery. This is the reason why, according to some authors, a direct transition from open surgery to the single-port approach could be simpler than moving from open surgery to multiportal VATS (11).

This geometrical understanding helped the technique become reproducible. Once surgeons realized that uniportal VATS required its own operative logic rather than simple adaptation of multiportal steps, the procedure could be standardized, taught, and disseminated.

### 3.3 The path towards reproducibility: standardizing the uniportal VATS technique

After the first cases, the next major phase was standardization. The technique had to be reproducible beyond a single surgeon or a single center. This required a precise definition of patient positioning, incision placement, camera handling, instrument arrangement, dissection sequence, stapling strategy, lymphadenectomy, and conversion criteria (12, 13). Standardization of the procedure was also possible due to the evolution of surgical instruments. The development of articulated staplers and curved instruments with both proximal and distal articulation has helped to achieve that internal crossing of surgical instruments necessary for major pulmonary resections through a single incision approach.

Sihoe's technical work was particularly significant in this process (12). He stressed that the patient should be positioned in full lateral decubitus, as in conventional VATS/open surgery, but considering that uniportal VATS lobectomy has an anterior approach, the surgeon should stand anteriorly with the main monitor placed opposite. This configuration permits straight operative axis from the surgeon to video-camera, through the wound, to the monitor. The incision is generally placed in the fifth intercostal space between the anterior and midaxillary lines. If the incision is too high, stapling angles to the hilar vessels may become unsafe; if it is too low, instrument fencing and excessive distance to the hilum may compromise dissection.

**Figure 1:**
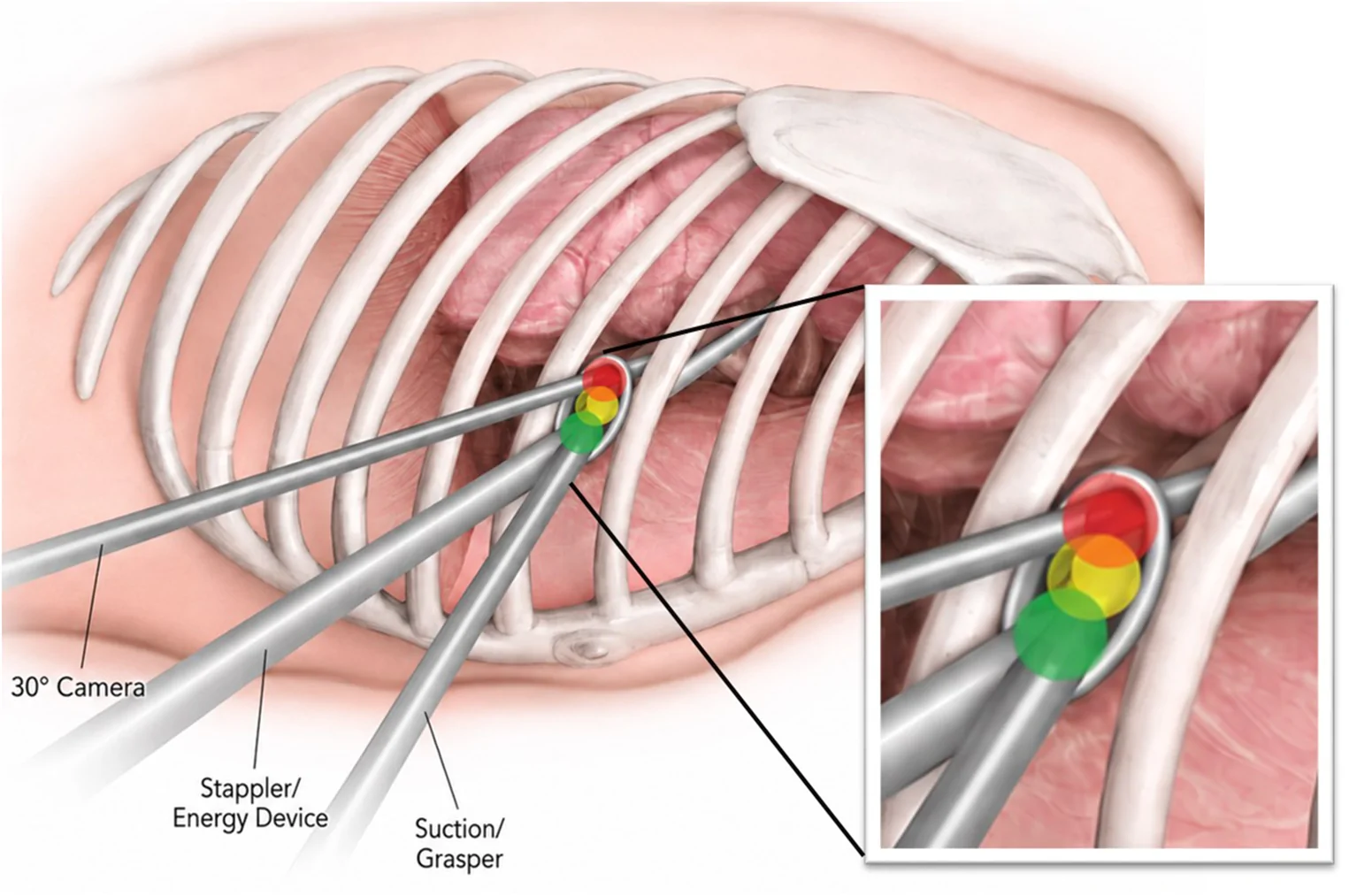
Traffic-light configuration. Schematic representation of the position of the camera and operative instruments during uniportal VATS. The camera is positioned in the upper and posterior part of the incision (red), whereas the energy device or stapler is placed in the lower part (green). The suction device or grasper, generally handled with the non-dominant hand or by the assistant, is positioned in the middle (yellow).

The “traffic-light” configuration became one of the most recognizable ergonomic rules of uniportal lobectomy. The camera is kept in the upper part of the incision (red light), while the surgeon's instruments work below it, generally with the grasper in the middle (yellow light) and the energy device or stapler in the bottom part (green light) (Figure 1). This configuration maintains the visual field above the operative instruments, reduces collision, and reproduces the relationship between eyes and hands in open surgery.

Standardization also involved adapting lobectomy strategies to the uniportal view. Lower lobectomies were initially considered more accessible, particularly when the fissure was complete and the pulmonary artery could be approached easily. Upper lobectomies required more refined stapling angles, especially for the truncus anterior and upper pulmonary vein. Fissureless techniques became important in patients with incomplete fissures, especially in right upper lobectomies to reduce the incidence of middle lobe impairment (14).

With increasing experience, the technique gained popularity, and many surgeons shifted from multiportal to uniportal VATS or directly from open surgery to the uniportal approach. Although no universal cut-off has been established, proficiency in uniportal VATS lobectomy is generally reported after approximately 30–50 cases, with a shorter learning curve for surgeons already experienced in multiportal VATS.

### 3.4 Global dissemination: from a single European center to a global surgical movement

The worldwide diffusion of uniportal VATS technique is one of the most distinctive characteristics of its history. In fact, most of the surgical innovations slowly spread thanks to randomized trials or industry-driven implementation, while uniportal VATS has expanded rapidly through a combination of published evidence, live surgery, international traineeship, hands-on courses and high-volume institutional experience.

From its starting point, the technique progressively moved from a single-surgeon practice to an international surgical movement. Europe played a central role not only in the birth of the technique, but also in its early institutional adoption. After the initial La Coruña experience, several European centers began structured programs of uniportal VATS lobectomy and reported their learning curves (11, 15, 16). The Papworth experience in the United Kingdom showed that uniportal VATS lobectomy could be safely performed by surgeons already experienced in multiportal VATS, with an acceptable learning curve and reproducible outcomes (16). One of the factors that most contributed to the rapid spread of this technique was the organization of masterclass and courses by uniportal VATS surgeons (17–19), especially by the pioneer Dr Gonzalez-Rivas.

In this context, the ultra-high-volume center of Shanghai Pulmonary Hospital has played a pivotal role in the global dissemination of uniportal VATS. Since 2013, the institution has hosted a two-week international training program that combines daily observation of more than 50 uniportal VATS procedures with animal hands-on practice, attracting approximately 100 surgeons annually from different parts of the world. In 2016, the outcomes of this educational program were reported, showing that most attendees improved their technical confidence and subsequently introduced the uniportal VATS approach in their own institutions (20).

Asia rapidly became one of the most important regions for validation, refinement, and technical expansion. Many Asian centers embraced uniportal VATS approach, and their ultra-high surgical volume allowed rapid accumulation of experience. Yu and Ng described Asia as a proving ground for single-port VATS, highlighting its spread across Taiwan, Korea, Hong Kong, Singapore, mainland China, and Japan, and emphasizing the contribution of Asian surgeons to technical refinements and specific instruments (21, 22). To date, Asia, and particularly China and Japan, is probably one of the regions where uniportal VATS has achieved the most widespread and capillary diffusion (23).

The Americas followed a different but equally relevant pathway. In Latin America, diffusion was strongly supported by training courses and regional educational initiatives (24–26). The São Paulo experience reported the development of uniportal VATS from diagnostic procedures to lobectomy, with 454 uniportal thoracic procedures performed between 2012 and 2016 with good results (26). In North America, uniportal VATS diffusion was more cautious, probably because multiportal VATS and robotic surgery were already well established. Nevertheless, institutional series from Canada and the United States later demonstrated that uniportal VATS could be incorporated safely into mature minimally invasive thoracic programs, with outcomes comparable to multiportal VATS (27). Recently, the group of Pittsburgh (PA, USA) published their first experience in uniportal VATS lobectomy, showing how the diffusion of this technique in North America is going slowly but progressively (28).

The African experience also illustrates how the technique could spread through educational training programs in countries with emerging economies. A report from South Africa described the first uniportal VATS wet lab in the country, involving four university centers, live surgery, lobectomies, and other thoracoscopic procedures. The initiative led to the introduction of thoracoscopic programs in all four participating centers, with plans to extend the program to other centers and countries (29). Moreover, the dissemination of uniportal VATS technique was possible thanks to global surgical training and transfer of expertise, including the use of a mobile thoracic surgery unit to deliver advanced minimally invasive procedures and hands-on education in resource-limited settings. However, to date, the dissemination of uniportal VATS in Africa remains limited and heterogeneous, mainly because of restricted access to dedicated equipment and lack of high-volume thoracic surgery centers.

### 3.5 Minimizing invasiveness: uniportal VATS segmentectomies, non-intubated, tubeless and subxiphoid approach

Recently, the increasing detection of small peripheral ground-glass nodules and the ageing population have created a renewed interest in anatomical sublobar resection. In 2022, the JCOG0802/WJOG4607L trial showed an advantage in overall survival for segmentectomy compared with lobectomy in patients with clinical stage IA NSCLC smaller than 2 cm and consolidation-to-tumor ratio >0.5, although local recurrence was higher after segmentectomy (30). This study firstly questioned the dogma that lobectomy is the gold standard treatment for early-stage NSCLC, suggesting that segmentectomy could be a viable option for lung cancer smaller than 2 cm and consolidation-to-tumor ratio >0.5. Complementary results were independently demonstrated by the CALGB/Alliance 140,503 trial (31). In their study, sublobar resections, including both segmentectomy and wedge resection, were non-inferior to lobectomy for disease-free survival, with similar overall survival, in patients with peripheral stage IA NSCLC ≤2 cm.

These trials contributed to the increasing clinical relevance of parenchyma-sparing resections and to the recognition of segmentectomy as an oncologically valid alternative in selected patients. The rise of uniportal VATS segmentectomy occurred in parallel with this growing oncological interest. In this context, performing segmentectomy through a small single thoracoscopic incision became an attractive goal. Considering the technical complexity of uniportal VATS segmentectomy and the greater anatomical variation, early reports were focused on relatively straightforward resections, such as lingulectomy, superior segmentectomies, and S6 segmentectomy. With increasing experience, the approach expanded to more complex procedures, including S3, S8, S9, S10, and combined or subsegmental resections (32).

Recent literature supports the feasibility of uniportal VATS segmentectomy in experienced centers. The first high-volume series were published by Duan et al., including their 2-year experience with the first 156 patients (33). Zhou et al. compared the perioperative and oncological outcomes of uniportal and three-port thoracoscopic segmentectomy in a large series of patients and found no difference (34). Moreover, Ahn and colleagues reported that short-term outcomes of uniportal VATS complex segmentectomy were comparable to those of simple segmentectomy, supporting its use as a safe and feasible option (35). Technological advances could help to manage difficult anatomy: three-dimensional CT reconstructions could better define segmental anatomy, bronchovascular variants, tumor location, and surgical margins while indocyanine green fluorescence has improved the identification of intersegmental plane (36).

Currently, the spread of segmentectomy for early-stage lung cancer appears to be the most logical evolution of uniportal VATS, combining a minimally invasive approach with a parenchyma-sparing surgery.

This mindset includes the integration of uniportal VATS with non-intubated, awake and tubeless thoracic surgery: once the extent of surgical resection had been reduced, it became logical to consider whether anesthetic and postoperative invasiveness could also be reduced. Non-intubated VATS aims to avoid tracheal intubation, neuromuscular blockade, and one-lung mechanical ventilation whereas awake surgery implies absent or minimal sedation with preserved patient cooperation. Potential advantages include reduced anesthetic drugs, less airway trauma, fewer ventilation-related complications and faster recovery (37, 38). Early experiences demonstrated the feasibility of non-intubated thoracoscopic wedge resection (39). Later, reports extended the technique to uniportal VATS anatomical pulmonary resections (40). Meta-analyses comparing non-intubated and intubated VATS for minor and major pulmonary resections suggest that non-intubated approach is feasible and may reduce postoperative pain, complications, chest-tube duration, and length of hospital stay (41, 42). However, non-intubated uniportal anatomical resections are technically and anesthesiologically demanding and evidence remains strongly influenced by patient selection and institutional expertise.

The ultimate evolution in reducing surgical, anesthetic and postoperative invasiveness is the “tubeless” strategy, defined as absence of orotracheal intubation and postoperative chest drains. This approach has been reported as feasible in carefully selected thoracic procedures and may facilitate early mobilization, reduced pain, shorter hospital stay, and even day-surgery pathways. However, the omission of a chest tube requires strict intraoperative air-leak control and postoperative monitoring to avoid unseen complication (43). Moreover, intraoperative cough, diaphragmatic movement, hypercapnia, dense adhesions, bleeding, difficult airway, or oncologically complex resections may require conversion to intubated general anesthesia that could be challenging in the lateral decubitus position. This strongly limits its widespread adoption outside selected experienced centers.

Similarly, the uniportal subxiphoid VATS approach faces comparable challenges. This could be considered a further evolution of minimally invasive thoracic surgery aimed at completely avoiding intercostal nerve injury. This approach has been successfully applied to wedge resections, segmentectomies, lobectomies and even more complex procedures with good results in terms of reduced postoperative pain and paresthesia, improved cosmetic results, and the possibility of accessing both pleural cavities through the same incision (44–46). However, the available evidence remains heterogeneous and is largely derived from retrospective studies and selected patient populations (47, 48). Moreover, the longer distance to the pulmonary hilum, the restricted stapling angles, the instrument interference, the need for dedicated instrumentation, the cardiac compression and the difficulty in achieving rapid control of major bleeding, increase the complexity of this technique and limit its adoption to a small number of experienced centers.

### 3.6 Expanding the boundaries: from standard resections to complex bronchovascular reconstructions

Once standard lobectomy and segmentectomy became reproducible, the next step was to test uniportal VATS for complex resections, traditionally reserved for thoracotomy. In fact, the need for bronchoplasty or vascular reconstruction was considered a contraindication to minimally invasive surgery for many years due to technical difficulty, precise suturing and immediate management of complications. In 2002, Santambrogio et al. (49) reported the first multiportal VATS sleeve lobectomy. However, its adoption and development in the following years was limited (50). In 2013, Gonzalez-Rivas and colleagues (51) reported the first bronchial sleeve lobectomy through uniportal VATS. The procedure was a right upper sleeve lobectomy performed using a 5-cm incision without rib spreading, including a complete lymphadenectomy, and an end-to-end bronchial anastomosis using thoracoscopic suturing.

The next major advance was pulmonary artery reconstruction. Vascular reconstruction is particularly challenging even in open surgery because it requires safe clamping, proximal and distal control and management of massive bleeding. The uniportal approach for pulmonary artery reconstruction was first reported by Gonzalez-Rivas et al. in a patient with an advanced left upper lobe tumor (52).

The uniportal technique then expanded to double-sleeve resections, combining bronchial and vascular reconstruction, and to carinal and tracheobronchial procedures. These operations require advanced thoracoscopic suturing, vascular control strategies, experienced anesthesia, immediate availability of conversion, and a team familiar with complex minimally invasive thoracic surgery.

While the initial experience was almost limited to a single-surgeon activity, as experience increased, uniportal sleeve lobectomy and pulmonary artery reconstruction were performed in different centers worldwide (53–56). Moreover, uniportal VATS was gradually applied also to selected advanced and complex cases such as post-induction therapy, tenacious adhesions, central tumors and Pancoast tumors.

The historical significance of these complex cases is not that every center should perform them through a single incision. Authoritative reports and technical reviews suggest that uniportal advanced cases should not be generalized to all units but must be limited to experienced uniportal VATS surgeons (57, 58). However, they demonstrated that the uniportal approach was not intrinsically limited to simple resections, but it could reproduce the principles of open surgery: exposure, vascular control, airway reconstruction, lymphadenectomy, and oncological radicality. Appropriate selection of the patients remains essential: uncontrolled bleeding risk, extensive chest wall invasion, inability to obtain vascular control, or surgeon discomfort should lead to a prompt conversion to open surgery. This remains a mandatory principle: the uniportal approach should expand only as far as allowed by patient safety, surgeon experience, and oncological quality.

### 3.7 Oncological feasibility and postoperative outcomes

The oncological feasibility of uniportal VATS has become a major focus of recent investigations. An increasing body of evidence suggests that, when performed by experienced surgeons, uniportal VATS provides oncological outcomes comparable to those achieved with conventional multiportal VATS. Comparative series have shown that uniportal and multiportal VATS achieve similar rates of complete resection and mediastinal lymph-node assessment, suggesting that the single-incision approach does not compromise surgical radicality (27). Available survival analyses also report comparable outcomes. Nachira et al. observed no significant differences between uniportal VATS and thoracotomy in 3-year overall survival (78% vs. 74%, *p* = 0.204), disease-specific survival (97% vs. 89%, *p* = 0.371), or 3-year disease-free survival (62% vs. 66%, *p* = 0.917) (59). Propensity-matched comparisons between uniportal and multiportal VATS similarly demonstrated comparable medium- and long-term overall and disease-free survival, without evidence that the surgical access independently affected recurrence or mortality (60, 61). A recent meta-analysis of propensity-score-matched cohorts reported comparable disease-free survival and no statistically significant difference in overall survival up to 96 months, although the available studies were heterogeneous and predominantly retrospective (62). More recently, the increasing use of neoadjuvant chemo-immunotherapy has introduced additional technical challenges, including dense hilar fibrosis, tissue fragility, and adhesions that may complicate vascular and bronchial dissection during minimally invasive procedures. Available series suggest that minimally invasive anatomical resection, including uniportal approaches in selected patients, remains feasible and safe in experienced centers (63). Overall, current evidence suggests that uniportal VATS can provide oncological outcomes comparable to multiportal VATS or thoracotomy. Nevertheless, robust multicenter prospective or randomized studies with standardized staging, recurrence definitions, and long-term follow-up remain limited.

Similarly, although comparative studies and meta-analyses generally suggest lower early postoperative pain and analgesic requirements than with multiportal VATS (62, 64), the available evidence is largely derived from retrospective studies, while randomized controlled trials remain scarce and have not consistently demonstrated a postoperative advantage of uniportal VATS (65, 66).

Systematic lymph node dissection was another central issue. One of the earliest criticisms of uniportal VATS was that the single incision might compromise mediastinal lymphadenectomy not allowing the exposure of all mediastinal stations. The La Coruña series reported a mean of 14.5 lymph nodes removed and a mean of 4.6 nodal stations explored, suggesting that oncologically appropriate nodal assessment was feasible through the uniportal approach (8). Subsequent experience from different centers worldwide also supported the feasibility of complete lymphadenectomy using single-port access (67). More recently, Mi et al. (68) reported that uniportal VATS approach was associated with a significantly higher number of dissected lymph nodes than three-port VATS lobectomy (13 vs. 9 nodes, *P* = 0.007), while the number of dissected lymph node stations was comparable between groups. These findings suggest that, in experienced hands, uniportal VATS provides lymphadenectomy quality comparable to conventional multiportal VATS, while no superiority of either technique can be assumed.

### 3.8 Redefining surgical paradigms: the influence of uniportal VATS on robotic thoracic surgery

The global spread of uniportal VATS also influenced RATS. Robotic approach presents several benefits such as improved three-dimensional visualization, greater maneuverability, use of bipolar energy and grasping instruments on both hands, wristed instruments and tremor filtration. However, the high costs limit its spread to selected hospitals. Traditional robotic-assisted thoracic surgery developed as a multiport technique, using generally 3–4 robotic arms and an assistant port (69).

As uniportal VATS became more widely adopted, surgeons began to explore whether the same single-incision idea could be moved to RATS. Yang Y and colleagues (70) performed the first uniportal RATS lobectomy in 2021, combining robotic dissection with conventional thoracoscopic stapling. Subsequently, González-Rivas and colleagues introduced the first pure uniportal RATS procedures (71), defining a standardized technique for port placement in single-incision robotic technique for anatomical lung resections. Since then, the idea of combining the advantages of the uniportal approach and the robotic platform has spread, with early experience showing good results (72–74). Recently, comparison studies showed that uniportal RATS is a safe and feasible approach with outcomes that are comparable to the other minimally invasive approaches even in complex cases (75, 76). Technological advances enabled the first transcontinental single-port RATS procedure in 2025 (77), during which a right upper lobectomy was performed by a lead surgeon located more than 8,000 km away.

The influence of uniportal VATS on uniportal RATS is therefore revolutionary. Uniportal VATS changed the culture of thoracic surgery by proving that major lung resections could be performed through one small intercostal incision. Uniportal robotic surgery represents an attempt to adapt this mindset into the robotic field. In recent years, efforts have been made to create specific single-port robotic platforms. Initial experiences with the Da Vinci single-port (SP) system (Intuitive Surgical, Inc., Sunnyvale, CA, USA) for subcostal anatomical lung resection in Asia and the United States demonstrated low conversion rates, satisfactory maneuverability, and favorable perioperative outcomes despite the relatively limited number of patients (78, 79). Similarly favorable results have been reported in China with the novel SHURUI Single-Port Serpentine-Arm Robotic Surgery System (Beijing Shurui Technology Co., Ltd.), achieving a 100% non-conversion rate, with only one case requiring an auxiliary port and a surgeon satisfaction rate of 95% (80, 81). Notably, this platform eliminates the need for articulated arm joints thanks to its “deformable dual continuum mechanism,” allowing its use through the conventional uniportal lateral approach.

## 4 Limitations

This narrative review has several limitations that should be acknowledged. First, its aim was to describe the evolution and progress of the uniportal VATS technique over the years in a narrative rather than systematic manner; therefore, study identification and selection were not based on a predefined systematic protocol, and selection bias cannot be excluded. Second, the available evidence on oncological and long-term outcomes is largely based on retrospective, single-centre series and reports from pioneering groups, which may limit the generalizability. Finally, some of the authors have directly contributed to the development and dissemination of uniportal VATS, which may introduce interpretative bias, although the review also incorporates evidence and perspectives from multiple international groups worldwide.

## 5 Conclusion

In conclusion, the evolution of uniportal VATS has represented far more than a technical refinement in minimally invasive thoracic surgery: it has marked a paradigm shift in the minimally invasive surgical treatment of lung cancer. Beyond redefining current standards of minimally invasive lung resection, uniportal VATS has also influenced the future direction of thoracic surgery itself. Many emerging technologies, including dedicated uniportal robotic platforms, are already moving towards the uniportal approach. Others, such as articulated VATS instruments and wireless camera systems, may further overcome current technical limitations and increase procedural accessibility.

Although uniportal VATS is now an established minimally invasive option for anatomical lung resection, future research, including multicenter prospective studies, long-term oncological outcomes, cost-effectiveness analyses, and learning-curve assessment, will be important to provide stronger evidence-based validation of its role.

As technological innovation converges with accumulated surgical expertise, uniportal approaches are likely to continue redefining the boundaries of minimally invasive thoracic oncology and consolidating their role among the standard approaches to minimally invasive thoracic surgery.
